# XBP1 Modulates the Aging Cardiorenal System by Regulating Oxidative Stress

**DOI:** 10.3390/antiox12111933

**Published:** 2023-10-30

**Authors:** Ji Zhang, Yuanyuan Zhao, Nianqiao Gong

**Affiliations:** 1 Anhui Province Key Laboratory of Genitourinary Diseases, Department of Urology, The First Affiliated Hospital of Anhui Medical University, Institute of Urology, Anhui Medical University, Hefei 230022, China; zhangji@alumni.hust.edu.cn; 2 Key Laboratory of Organ Transplantation of Ministry of Education, Institute of Organ Transplantation, Tongji Hospital, Tongji Medical College, National Health Commission and Chinese Academy of Medical Sciences, Huazhong University of Science and Technology, Wuhan 430030, China; yyzhao@tjh.tjmu.edu.cn

**Keywords:** XBP1, aging, cardiorenal system, oxidative stress

## Abstract

X-box binding protein 1 (XBP1) is a unique basic-region leucine zipper (bZIP) transcription factor. Over recent years, the powerful biological functions of XBP1 in oxidative stress have been gradually revealed. When the redox balance remains undisturbed, oxidative stress plays a role in physiological adaptations and signal transduction. However, during the aging process, increased cellular senescence and reduced levels of endogenous antioxidants cause an oxidative imbalance in the cardiorenal system. Recent studies from our laboratory and others have indicated that these age-related cardiorenal diseases caused by oxidative stress are guided and controlled by a versatile network composed of diversified XBP1 pathways. In this review, we describe the mechanisms that link XBP1 and oxidative stress in a range of cardiorenal disorders, including mitochondrial instability, inflammation, and alterations in neurohumoral drive. Furthermore, we propose that differing degrees of XBP1 activation may cause beneficial or harmful effects in the cardiorenal system. Gaining a comprehensive understanding of how XBP1 exerts influence on the aging cardiorenal system by regulating oxidative stress will enhance our ability to provide new directions and strategies for cardiovascular and renal safety outcomes.

## 1. Introduction

As research efforts have intensified, a complex hemodynamic and neurohumoral connection between the heart and the kidneys has become highly evident [1,2]. Even in the absence of damage, the glomerular filtration rate (GFR) is known to decrease by approximately 8 mL/min/1.73 m^2^ per decade after the age of 40 years [3]. Furthermore, research has shown that the incidence of acute kidney injury (AKI) is higher in older adults [4]. The progression of kidney damage leads to an expansion of blood volume, along with an upregulation of the sympathetic nervous system (SNS) and the renin–angiotensin–aldosterone system (RAAS); these changes exert numerous maladaptive systemic effects on the heart, vasculature, and kidneys [5,6,7,8]. Compared to young hearts, aged hearts are more susceptible to both acute and chronic damage [9]. Aged hearts have little protection against such injury [10]; thus, heart failure is a typical disease related to the elderly [11]. Consequent venous congestion, inadequate renal blood flow, and poor renal perfusion result in renal dysfunction attributed to a decline in renal blood flow and glomerular filtration rate (GFR), along with reduced urinary output [12,13,14]. In addition, these changes elevate the activity of SNS and the release of renin from the juxtamedullary apparatus, thus resulting in sodium retention and enhanced vascular congestion, further exacerbating heart failure [8,14]. In a previous study, Claudio et al. defined five types of cardiorenal syndromes based on pathophysiology, time frame, and the presence or absence of cardiac and renal dysfunction. Types 1 and 2 are caused by acute heart injury and chronic heart failure, respectively, while types 3 and 4 are caused by AKI and chronic kidney disease (CKD), respectively. Type 5 is caused by other systemic diseases such as sepsis and diabetes [15].

**Figure 1:**
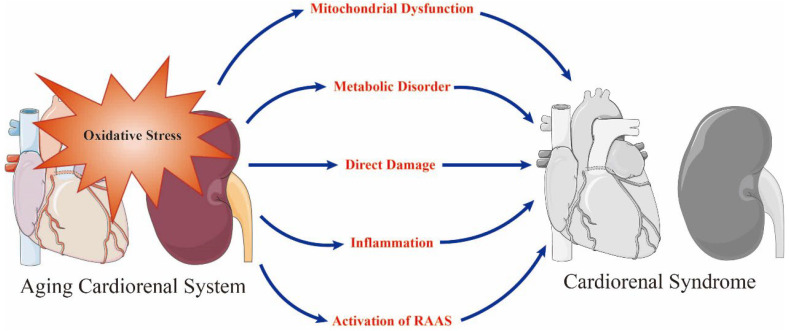
Oxidative stress is a prominent initiator of mitochondrial dysfunction, metabolic disorder, direct damage, inflammation and the activation of renin–angiotensin–aldosterone system (RAAS) in the aging cardiorenal system and plays a key role in the occurrence of cardiorenal syndrome. Adobe Illustrator (version 2021 25.0) was used for the production of this figure.

Although a variety of factors can cause senescence-related cardiorenal interactions, a strong body of evidence indicates that oxidative stress plays a central role [14,16] via age-induced metabolic, hemodynamic, neurohormonal, and inflammatory mechanisms, and atherosclerotic degeneration [17,18,19]. Aging is highly correlated to the process of mitochondrial dysfunction and causes the accumulation of oxidative stress in cardiomyocytes and nephrocytes [17,20,21]. Furthermore, oxidative stress participates in the generation of biomarkers that can be used to detect cardiorenal aging and functional deterioration, including brain natriuretic peptide (BNP)/N-terminal (NT)-pro hormone BNP (NT-proBNP), interleukin-1β (IL-1β), nicotinamide adenine dinucleotide phosphate (NADPH) oxidases (NOX), NLR family pyrin domain containing 3 (NLRP3) and neutrophil gelatinase associated lipocalin (NGAL) [7,22]. In addition to stimulating SNS and RAAS, oxidative stress weakens cardiorenal function by attenuating the restoration of mitochondrial health, alleviating mitochondrial biogenesis, enhancing proinflammatory and profibrotic pathways, and damaging the integrity and viability of cells and organs [6,7,8] (Figure 1).

**Figure 2:**
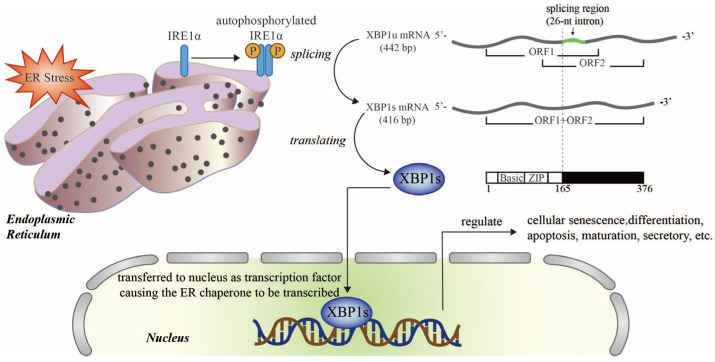
Schematic diagram of the XBP1 signaling pathway in endoplasmic reticulum stress (ER stress). Upon ER stress, IRE1α is auto-phosphorylated and transformed into an active dimer which splices XBP1u mRNA into XBP1s mRNA, which codes for an active transcription factor, XBP1s. The translocation of XBP1s to the nucleus promotes the transcription of target genes that regulate senescence, survival, metabolism, and the immune system. Ⓟ Refers to phosphoric acid. Adobe Illustrator (version 2021 25.0) was used for the production of this figure.

Cardiorenal abnormalities contribute to the accumulation of misfolded or unfolded proteins in the endoplasmic reticulum (ER), thereby exerting significant load on the ER protein-folding mechanism; this can overwhelm the capacity of the ER, a disease state known as ER stress [23,24]. Cells have evolved an adaptive signal transduction pathway that transmits signals from the ER to the nucleus, known as the unfolded protein response (UPR); this pathway attempts to restore ER homeostasis and improve cellular functional recovery against ER stress [25]. X-box binding protein 1 (XBP1), a member of the cAMP response element binding protein (CREB)/activating transcription factor (ATF) basic region-leucine zipper family of transcription factors, is one of the highly conserved effector molecules of UPR [26]. Upon ER stress, inositol requiring enzyme 1α (IRE1α) splices *XBP1* pre-mRNA to *XBP1* mRNA, which is then translated into an active transcriptional factor (XBP1s; the spliced form of XBP1), thus regulating the transcription of UPR genes encoding ER chaperones that are closely related to physiological and pathological activities such as cell death, regeneration, and metabolism [25] (Figure 2). XBP1 has been demonstrated to be expressed ubiquitously, from yeast to humans [27,28,29,30,31,32,33]. The complete knockout of XBP1 was previously shown to result in embryonic lethality [34,35,36,37]. We previously investigated the effects and mechanisms that control homeostasis in the heart and kidneys [8,22,25,26,38,39,40] and found that oxidative overload is likely to induce or prevent *XBP1* mRNA splicing. Furthermore, the efficient activation of XBP1 exerts protective effects on oxidative injury and helps to sustain the viability of the cardiorenal system and the integrity of cardiorenal function [6,41,42]. In contrast, aging increases the risk of XBP1 being excessively activated; this condition is closely associated with mitochondrial dysfunction and the overproduction of free radicals via a range of mechanisms including calcium imbalance, the initiation of ER biogenesis, and ER-dependent apoptosis [43,44,45]. Herein, we review the characteristics of these biomarkers and pathways in the aging cardiorenal system and the contributions of XBP1s, thus identifying potential targets to develop much needed novel therapeutic concepts against cardiorenal syndrome in the elderly.

## 2. Mitochondrial Maintenance

Mitochondria generate more than 90% of adenosine triphosphate (ATP); this represents approximately one-third of the high energy demand of cardiomyocytes; consequently, the human heart contains the highest concentration of mitochondria in the entire body [46]. Owing to the high energy demands of solute reabsorption, the kidneys, particularly cells in the proximal tubules and the medullary thick ascending limb, possess an abundance of mitochondria [47,48]. Mitochondria are bioenergetic and biosynthetic signaling organelles that provide a critical stress-sensing function that helps cells to adapt to their environment [49]. Over the past few decades, researchers have firmly concluded that the mitochondrion is a central contributor to oxidative stress in the cardiorenal system [22,39,50,51,52]. XBP1 is often implicated in many cardiorenal diseases as consequences of failed mitochondrial maintenance [22,26].

In both healthy kidneys and hearts, physiological levels of mitochondrial reactive oxygen species (ROS) can activate survival pathways [53]. When the heart experiences biological stressors, such as myocardial ischemia-reperfusion injury (IRI), there is a marked reduction of mitochondrial membrane potential (ΔΨm) and ATP stores become exhausted; these changes are accompanied by acidosis that is secondary to lactate accumulation, and an increase in the intracellular calcium (Ca^2+^) concentration. Furthermore, the outer mitochondrial membrane (OMM) remains intact and the mitochondrial permeability transition pore (mPTP) remains closed. With subsequent reperfusion, the reintroduction of oxygen leads to the rapid normalization of pH and a rapid restoration of ΔΨm, thus precipitating a range of adverse sequelae including the production of mitochondrial ROS, the exacerbation of Ca^2+^ overload, OMM destruction, and mPTP formation [54].

It is well established that acute kidney injury (AKI) perturbs the usual vectorial pumping of protons across the inner mitochondrial membrane by enzymatic complexes within the mitochondrial electron transport chain (ETC) [55]. The subsequent loss of ΔΨm disrupts selective permeability. As a consequence, the mitochondria expand [56]. Mitochondrial biogenesis refers to the cellular process by which new mitochondrial mass and mitochondrial DNA (mtDNA) replication are produced [57], enhancing mitochondrial oxidative phosphorylation (OXPHOS) capacity and the repair of mitochondrial dysfunction after AKI. Mitochondrial fusion joins two mitochondria at the outer and inner membrane interfaces via several membrane GTPases, Mitofusin 1 (MFN1), Mitofusin 2 (MFN2), and Optic atrophy protein 1(OPA1) [58,59]. Mitochondrial fission is a multistep process that allows a mitochondrion to split in two separate mitochondrial organelles [60]. The network formed by mitochondrial fusion–fission is mitochondrial dynamics. Mitochondrial dynamics is deployed to sequester damage and efficiently eliminate damaged mitochondria through mitophagy, which is a mechanism that selectively degrades excess and defective mitochondria [61,62]. It is worth noting that changes in the mitochondrial structure have been observed in the ischemic human kidney prior to the occurrence of the clinical symptoms of AKI, thus suggesting that mitochondrial perturbation may not be a mere epiphenomenon following injury. Rather, mitochondrial dysfunction could potentially be a factor that contributes to injury [63].

In addition, heart failure and CKD also induce mitochondrial biosynthesis, mitochondrial dynamics, mitophagy, and mitochondrial proteostasis; collectively, these processes facilitate mitochondrial quality control (MQC).

**Figure 3:**
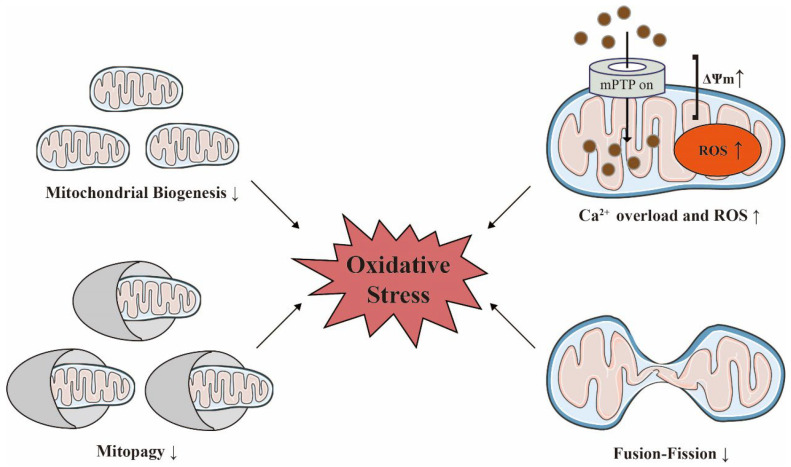
Mitochondrial homeostasis is decisive for maintaining oxidative stress with age. Acute injuries can trigger an increase in mitochondrial membrane potential (ΔΨm), calcium overload, and the overproduction of reactive oxygen species (ROS) in damaged mitochondria. The reduced functionality of biosynthesis, dynamics, and quality control in heart failure and chronic kidney disease can also exacerbate oxidative stress. The ↑ indicates an increase; the ↓ indicates a decrease. Adobe Illustrator (version 2021 25.0) was used for the production of this figure.

It is thought that appropriate MQC is a compensatory molecular mechanism that removes anomalous mitochondrial proteins or completely damaged mitochondria, and restores mitochondrial both function and homeostasis, thus favoring cardiorenal protection [64,65]. In contrast, the disruption of MQC accelerates lethal damage [66]. Mitochondrial dysfunction and imbalances in MQC are hallmarks of the aging cardiorenal system [26,67]. These mitochondrial events driven by oxidative stress contribute to increased susceptibility of the aging kidneys and hearts to acute and chronic injuries [68]. Intriguingly, the specific ablation of mitochondria from senescent cells was previously shown to be sufficient to reverse many features of the senescent phenotype [69] (Figure 3).

### 2.1. Nuclear Factor Erythroid 2-Related Factor 2 (NRF2)

NRF2, encoded by *nuclear factor*, *erythroid 2 like 2* (*NFE2L2*) gene, is a master regulator of anti-aging and antioxidant defense and protects against various insult-induced organ damage [8,22,39,70,71] by regulating responses to mitochondrial-derived ROS via its pleiotropic effects on controlling antioxidant and detoxification genes, including NADPH-quinone oxidoreductase (NQO1), heme oxygenase-1 (HO-1), and superoxide dismutase 1 (SOD1) [70,71,72], preventing the progression of AKI to CKD transition and deeply participating in CKD development. AKI activates the expression of the NRF2 in the kidneys, thereby enhancing antioxidant target gene transcription that protects the kidney from oxidative damage [39]. Compared to healthy kidney tissue, NRF2 and its downstream molecules were found mainly upregulated in earlier human CKD samples, like glomeruli of diabetic nephropathy (DN) patients, and kidney tissue from patients with lupus nephritis [73,74]. Because of the imbalance of antioxidant mechanism, decreased expression of the NRF2 system is often detected in patients with advanced CKD [75]. The transcription of NRF2 can also increase mitochondrial biogenesis through the expression of genes that are essential for mitochondrial biogenesis, including nuclear respiratory factor 1 (NRF-1) and peroxisome proliferator-activated receptor γ co-activator 1α (PGC-1α) [76,77,78].

It has been well documented that the Kelch-like ECH-associated protein 1 (Keap1)/NRF2 and the glycogen synthase kinase-3β (GSK-3β)/NRF2 signaling pathways represent redox-sensitive regulator axes through which NRF2 dissociates from Keap1 or GSK-3β under oxidative stress prior to the induction of various antioxidant genes [22,79,80]. In addition to this quintessential mechanism, our laboratory provided evidence to indicate that XBP1s induces the transcriptional upregulation of hydroxymethylglutaryl reductase degradation 1 (HRD1) [22], an E3 ubiquitin ligase that is also known as synoviolin 1 (SYVN1) that coordinates ER-associated protein degradation (ERAD), a process that directly catalyzes ubiquitin conjugation onto unfolded or misfolded proteins for proteasomal degradation [8,22]. Furthermore, HRD1 can ubiquitinate and degrade NRF2 via interaction with the QSLVPDI motif, while the blockade of HRD1 can prevent the downregulation of NRF2 in tubular epithelial cells experiencing IRI [22]. Ferroptosis-associated epithelial and endothelial to mesenchymal transition (EMT) is the principal pathological basis underlying the progression of DN to end-stage renal disease (ESRD), which often involves the accumulation of ROS and iron overload in renal tubular epithelial cells. This is probably caused by activation of the XBP1-HRD1-NRF2 pathway by high levels of glucose, at least in part [81]. In line with this, investigations from another laboratory reported that the cardiac expression of XBP1s could effectively rescue the expression of HRD1 and mediate the ubiquitination and degradation of NRF2 [8].

### 2.2. O-Linked GlcNAc Modification (O-GlcNAcylation)

O-GlcNAcylation, a pro-survival pathway that counterbalances age-related decline in a self-healing capacity [82], can modulate protein stability and function, and has been implicated in various cardiovascular diseases. In cardiac IRI, XBP1s couples ER stress with the hexosamine biosynthetic pathway (HBP) by triggering activation of the major HBP enzymes: glutamine fructose-6-phosphate aminotransferase 1 (GFAT1), glucosamine-phosphate N-acetyltransferase 1 (GNPNAT1), phosphoglucomutase 3 (PGM3), and uridine diphosphate-glucose 4-epimease (GalE) [83]. The XBP1s-HBP axis promotes the synthesis of uridine diphosphate N-acetylglucosamine (UDP-GlcNAc), an obligate substrate for the O-GlcNAcylation of cardiac proteins during IRI [83,84]. O-GlcNAcylation has also been implicated in various IRI-related cardiovascular diseases. A body of evidence suggests that increasing O-GlcNAcylation during IRI may represent a unique and endogenously recruitable mechanism of cardioprotection that acts directly via O-GlcNAcylating voltage-dependent anion channel 1 (VDAC1) in the mitochondria [85,86,87]. Because VDAC is a putative form of mPTP, the O-GlcNAcylation of VDAC prevents the formation of mPTP, alters sensitivity to the loss of ΔΨm, relieves Ca^2+^ overload-induced mitochondrial swelling, and hence maintains mitochondrial stability [85].

### 2.3. BNP and NT-proBNP

Under pathological conditions, XBP1s, which is induced in response to ER stress, is an essential regulator of BNP transcription in cardiomyocytes, and binds to the proximal activator protein 1 (AP-1)/cAMP response element (CRE)-like element in the BNP promoter and increases the activity of its promoter [7,88]. The transcribed unstable *BNP* mRNA can rapidly synthesize a 134 amino acid BNP precursor (pre-proBNP) and remove the N-terminal 26 amino acid signal peptide to form a 108 amino acid BNP (proBNP) [89]. Subsequently, proBNP is cleaved into inactive NT proBNP (comprised of 76 amino acids) and active BNP (comprised of 32 amino acids) by proBNP convertases (corin or furin) [89,90,91]. Both BNP and NT-proBNP status are widely used as diagnostic biomarkers for heart failure, hypertension, and cardiac hypertrophy [89,92]. The significant interaction between changes in NT-proBNP and the reduction of GFR values indicate that NT-proBNP may precede the deterioration of renal function in patients with heart failure [93].

As a compensatory protection mechanism in the early stages of disease progression, BNP is a novel mitochondrial fusion activator, in addition to inducing natriuresis and dieresis, and reducing RAAS and SNS activity [43,94,95]. BNP binds to its receptor, the natriuretic peptide receptor-A (NPRA), activates protein kinase G (PKG), and then stimulates signal transducer and activator of transcription 3 (STAT3) by phosphorylation [95]. Phosphorylated STAT3 binds to the optic atrophy 1 (OPA1) promoter and promotes OPA1-mediated mitochondrial fusion [95]; this process protects against the cardiac dysfunction associated with mitochondrial depolarization and ROS production [43,95].

## 3. Inflammation

It is now well established that oxidative stress links XBP1 to inflammation [1,2,26]. The overexpression of proinflammatory cytokines may be linked to the pathogenesis of anemia in patients with heart failure. Inflammation can impact the function of endothelial kidney cells by causing exposure to a proinflammatory and prothrombotic profile, vasoconstriction, and capillary obstruction, thus resulting in AKI. Moreover, proinflammatory cytokines may reduce the production of red blood cells in the bone marrow by damaging red blood cell precursors and by limiting the expression of erythropoietin receptors; this process may result in anemia. The reduction of renal oxygen delivery due to the hypoperfusion of nephrons and low hemoglobin levels may affect aerobic metabolism in cells, thus resulting in cellular death [96,97]. Hence, oxidative stress can cause cardiorenal tissue inflammation, a process characterized by the activation of inflammatory cells and high circulating levels of inflammatory molecules; this process has been proposed as an oxidative stressor in acute and chronic cardiorenal impairment with advancing age [98,99,100,101]. In addition, proinflammatory and anti-inflammatory cytokines, along with chemokines, released by the kidneys can reach the circulation, thus leading to the dysfunction of the cardiovascular system via the main circulation [102].

### 3.1. NLRP3

Aging-related cardiovascular diseases and renal diseases are often associated with inflammasomes; these cytosolic multi-protein complexes, consisting of apoptosis-associated speck-like protein containing a caspase-associated recruitment domains (ASC), procaspase-1, inflammasome nucleators such as NLRs, AIM2, and pyrin, are responsible for innate immunity and involved in almost all cardiorenal diseases [103]. There are many types of inflammasomes; NLRP3 is the most well-characterized of these inflammasomes and acts as a receptor for cardiorenal damage, metabolic stress and ROS surveillance [104,105]. In addition, NLRP3 triggers the induction of cleaved caspase-1 which plays a role in pyroptosis and the release of cytokines belonging to the IL-1β family, primarily IL-1β and IL-18 [26,105]. In the clinical data of AKI and early-stage CKD, it is evident that the expression of NLRP3 component increases, indicating that NLRP3 guides the occurrence and prognosis of these diseases [106,107,108]. On the contrary, low levels of NLRP3 or caspase-1 were measured in some populations of lupus nephritis, urate nephropathy (UAN), and ESRD patients [109]. The changes in NLRP3 activation differs also between acute and chronic cardiac injury. Acute cardiac injury, such as cardiac IRI, is associated with remarkably increasing NLRP3 inflammasomes [110,111], while low basal activation of NLRP3 inflammosomes contributes to chronic cardiac diseases progression, including atherosclerosis, hypertensive heart disease, diabetic cardiomyopathy, and heart failure [111].

In our previous work, we found that NLRP3 was localized to both the mitochondria and the ER of tubular epithelial cells in the resting state. In addition, we found that the expression and activity of NLRP3 were elevated during renal IRI and were reduced by the knockdown of XBP1. Further experimental verification suggested that XBP1s could translocate into the nucleus to enhance NLRP3 gene promoter activity; this induced the increased clustering of NLRP3 inflammasomes on the mitochondria and the mitochondrial associated membranes (MAMs), eventually exacerbating caspase-1-dependent mitochondrial damage and the production of mitochondrial ROS in the kidneys exposed to IRI [26].

### 3.2. NOX and Nuclear Factor Kappa-B (NF-κB)

The NOX family of proteins represents the chief source of controlled ROS formation and includes seven isoforms with a broad tissue distribution and activation mechanism, including NOX1, NOX2, NOX3, NOX4, NOX5, and the dual oxidases (Duox1 and Duox2) [112]. NOX functionality and redox-based signaling play critical physiological and pathophysiological roles in aging [113]. NF-κB is an important nuclear transcription factor that plays a key role in all diseases characterized by inflammatory processes.

Recent studies have shown that hearts suffering from pressure overload exhibit increased NOX4 expression and ROS generation, thus resulting in the splicing of XBP1 and the activation of receptor interacting protein kinase 1 (RIPK1)-related NF-κB signaling downstream of XBP1s, ultimately leading to cardiomyocyte hypertrophy [7,114]. Correspondingly, XBP1s can also exacerbate lipopolysaccharide (LPS)-associated cardiomyocyte injury by downregulating the X-linked inhibitor of apoptosis protein (XIAP) and SOD by activating the NF-κB signaling pathway [115]. In human mesangial cells (HMCs), the silencing of XBP1 expression amplifies low-density lipoprotein (LDL)-induced inflammation via feedback based on the increased activity of the IRE1α/IκB kinase (IKK)/NF-κB pathway [100]. In the vasculature of spontaneously hypertensive rats (SHR), NOX4-related ROS provoked IRE1α oxidation which then accelerated the activation of XBP1s; this process improved the survival and proliferation of vascular smooth muscle cells (VSMCs) and improved the hypertension causing vascular dysfunction [116].

Macrophage-mediated innate and adaptive immune responses have been postulated as a notable mechanism during the pathogenesis of cardiorenal syndrome. Toll-like receptors (TLR) 2 and 4 are highly expressed by macrophages and respond to bioactive molecules secreted into the circulation due to cardiorenal tissue damage, such as the soluble form of biglycan (sBGN) [117,118]. Activated TLRs specifically upregulate XBP1s via NOX, thus facilitating the activation of NF-κB to elicit the release of proinflammatory cytokines, predominantly in macrophages. Interestingly, a recent study showed that TLR/NOX2-induced ROS was able to impair the expression and maturation of IL-1β [119]; furthermore, TLR/NOX1/4-induced ROS favors the expression and maturation of IL-1β in macrophages [120,121].

### 3.3. Transforming Growth Factor-β (TGF-β) 1

TGF-β1 has been identified as an essential regulator of the cardiorenal fibrotic process in the aging population [122,123,124]. After extensive or sustained inflammation, resident macrophages, myocardial cells, and renal parenchymal cells secrete TGF-β1 [125,126,127]; this process reduces the production of antioxidants and enhances oxidative stress, thereupon accentuating fibrosis by stimulating the production of extracellular matrix (ECM), activating resident populations of fibroblasts, and inducing EMT [128]. Furthermore, TGF-β1-induced fibrosis is known to primarily depend on TGF-β1/Smad signaling [127,129,130]. Tumor necrosis factor (TNF) receptor-associated protein 1 (TRAP1, also known as heat shock protein (HSP75), a member of the HSP90 chaperone family that resides principally in the mitochondria [131,132], is associated with the TGF-β/Smad signal transduction pathway [133]. XBP1s acts directly upstream of TRAP1; furthermore, the XBP1s-TRAP1 axis can inhibit the production of TGF-β1, prolong G2/M cell cycle arrest, reduce the expression of profibrotic factors, and ameliorate the progression of fibrosis [134].

### 3.4. Vascular Endothelial Growth Factor (VEGF) A

VEGFA is produced by most cells in the body, but is known to be upregulated in hypoxia. Furthermore, research has shown that VEGFA plays an important role in regenerative capacity via vasculogenesis and angiogenesis [135,136,137]; this requires ROS derived from NOX (especially NOX2 and NOX4) and gradually declines with age [138,139].

As a transcription factor, XBP1s binds to two regions on the VEGFA promoter and induces the upregulation of VEGFA in the heart and kidneys [140]. The XBP1s/VEGFA axis has been proven to be a novel regulatory pathway for vasculogenesis and angiogenesis [141] and can contribute to the progression of adaptive hypertrophy during heart failure. In addition, this axis can contribute to chronic inflammation and oxidative stress to induce diabetic cardiomyopathy [140]. During IRI, there is a reduction in renal VEGFA levels; however, research has shown that VEGFA is upregulated in both glomeruli (podocytes, endothelial cells, and mesangial cells) and in the tubular compartment during the early stages of diabetic kidney disease (DKD); in addition, VEGFA promotes subsequent alterations in vascular remodeling, inflammatory processes, glomerulosclerosis, and tubulointerstitial fibrosis [140,142,143,144].

## 4. SNS and RAAS

SNS influences intrarenal hemodynamics and stimulates RAAS via the juxtaglomerular apparatus of the kidney. Activation of the RAAS cascade typically commences with the secretion of prorenin and renin; this process induces pro-fibrotic effects in the kidneys by binding to the prorenin–renin receptor (PRR) [6,8]. The hypoperfusion of peripheral tissue in heart failure induces over-activity of SNS, thus resulting in the increased release of renin from the juxtamedullary apparatus. The synthesis of renin is also stimulated by the reduction of hydrostatic pressure in the glomerular afferent arterioles and a reduction in the amount of sodium delivered to the macula densa [7,145]. Subsequently, angiotensinogen (AGT) is converted into the decapeptide angiotensin I (AT1) by renin or the chymase enzyme. Then, AT1 is cleaved by angiotensin converting enzyme (ACE) to generate angiotensin II (AT2); this can lead to renal efferent arteriolar vasoconstriction and the formation of aldosterone, which increases tubular sodium reabsorption in the kidney and the effective circulating plasma volume by binding to either angiotensin II type 1 receptor (AT1R) or angiotensin II type 2 receptor (AT2R) [14]. ACE2, a homologue of ACE, acts on AT1 and AT2 to produce a 9-amino acid peptide known as angiotensin 1-9 (Ang1-9) and a 7-amino acid peptide known as angiotensin 1-7 (Ang1-7), which can protect hearts and kidneys against damage, fibrosis and remodeling by binding to angiotensin receptor type 2 or the endogenous orphan Mas receptor (MasR) [146]. Aged kidneys possess a lower abundance of cells of renin lineage and reduced responsiveness to RAAS inhibition [147]. One of the consequences of the RAAS system is an increase in the generation of ROS via NOX and increased protein aggregation, thus assisting the development of cardiorenal syndrome [8,14,148].

The XBP1s-HRD1-NRF2 axis exerts functional dichotomy in the cardiorenal system. When the ubiquitination/degradation functionality of the XBP1s-HRD1 axis is inhibited, NRF2 is aberrantly expressed and retained in the nucleus, thus resulting in the dysfunctional expression of RAAS genes, including the upregulation of AGT/ACE genes and the downregulation of ACE2/MasR genes; these effects can aggravate cardiorenal diseases [8,149]. XBP1s can also directly manipulate the expression of some genes that encode components of the RAAS. In human umbilical vein endothelial cells (HUVECs) treated with arsenite, XBP1s was responsible for the accumulation of hypoxia-inducible factor 1α (HIF1α) and the assembly of an XBP1s/HIF1α transcriptional complex, which occupies the responsive elements of the ACE/AT2/AT1R axis within the promoter region of the RAAS gene. This process attracts RNA polymerase II and drives transcription, thus causing the occurrence of oxidative stress and proinflammatory response by the activation of RAAS in several cardiovascular diseases, including hypertension, atherosclerosis, and microvascular abnormalities [6,7].

## 5. The Akt Pathway

Akt, also known as protein kinase B (PKB), is a crucial signaling protein that is strongly activated by cellular damage and governs cellular survival, proliferation, and senescence [24,150,151,152,153]. Many studies have demonstrated that Akt plays a significant role in protecting the aging cardiorenal system from oxidative stress [24,150,151,152]. The 78 kDa glucose-regulated protein (GRP78, also known as BiP/HSPA5), is a master protein chaperone located in the ER and modulates homeostasis in the ER [154]. As myocardial cells experience IRI, the XBP1s signaling branch and its downstream target GRP78 are activated in a highly robust manner. Subsequently, a fraction of GRP78 is translocated to the cell surface to stimulate the Akt-dependent suppression of ROS accumulation by interacting with phosphatidylinositol 3-kinase (PI3K) [150]. Previous DN research discovered that the overexpression of XBP1 in glomerular mesangial cells (MCs) exposed to high glucose (HG) could activate the phosphatase and tensin homolog deleted on chromosome ten (PTEN)/Akt signaling pathway, thus mitigating oxidative stress caused by HG or MCs apoptosis [24].

## 6. Myo-Inositol Oxygenase (MIOX)

MIOX, a cytosolic enzyme expressed predominantly in the renal proximal kidney tubules, catalyzes the conversion of myo-inositol to d-glucuronic acid and activates the glucuronate-xylulose pathway, which increases the ROS generation and can impose oxidative stress. It has been noted to be associated with tubulopathy in the context of DKD and AKI [23,155]. Sharma et al. reported that an increased nuclear translocation of XBP1s could bind MIOX promoter in the tubular compartment of diabetic mice, prompting the excessiveness of ROS via MIOX-mediated increased oxidant stress, which eventuated deleterious tubulointerstitial effects [23].

## 7. XBP1u

XBP1u, translated from *XBP1*, is an unspliced mRNA isoform and acts as a regulator with the same N-terminal and internal DNA binding domains as XBP1s. XBP1u consists of a basic region and a leucine zipper region [156]; in addition, the C-terminus of XBP1u contains a nuclear exclusion signal and lacks the transcription activation domain that is evident in XBP1s [157].

Most cardiorenal studies of XBP1u have focused on blood vessels [158,159,160]. In the aging cardiorenal system, pathological states involving disturbed flow can cause ligand-independent activation of the kinase insert domain receptor (KDR), which then upregulates XBP1u and histone deacetylase 3 (HDAC3) in endothelial cells [160]. Furthermore, because XBP1u can counteract XBP1s by sequestering XBP1s for proteasomal degradation, the overexpression of XBP1u has been shown to protect HDAC3 from the transcriptional repression caused by XBP1s [158,161]. XBP1u and HDAC3 promote the formation of the mammalian target of rapamycin complex 2 (mTORC2)-Akt1-XBP1u-HDAC3 complex and increases mTORC2-dependent Akt1 phosphorylation, which subsequently enhances the stability of NRF2 and NRF2-mediated HO-1 expression and reduces oxidative stress in endothelial cells during atherosclerosis and the formation of neointima post-injury [158,160].

## 8. Conclusions and Future Perspectives

**Figure 4:**
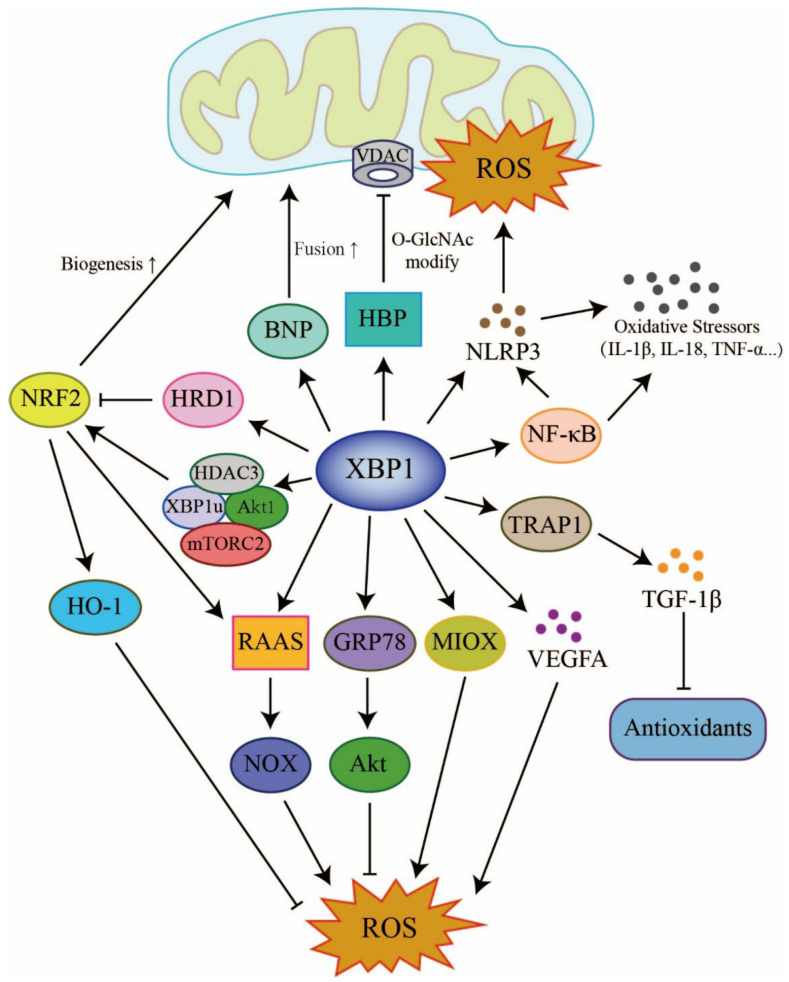
XBP1 orchestrates an intricate oxidative stress network via its role as a transcription factor and can control the expression of many molecules related to mitochondrial function and morphology, the immune system, the renin–angiotensin–aldosterone system (RAAS), and the antioxidant defense system. The ordinary arrow indicates the promotion; the T-shaped arrow indicates suppression. Adobe Illustrator (version 2021 25.0) was used for the production of this figure.

Existing literature indicates that XBP1 plays a critical role in the survival of senescent cells by coordinating the responses of the cytoplasm, ER and mitochondria to cellular stress, and alleviates the risk of age-related acute and chronic diseases [162]. However, there is a growing body of evidence to support a relationship between high expression levels of XBP1 and the induction of oxidative stress [8,22,26]; this relationship is indispensable for normal cellular activity but is also a causative factor of aging. The mechanisms responsible for the effect of XBP1 on cellular sensitivity to oxidative stress and aging have yet to be elucidated. Research has clearly shown that the heterogeneous pathways mediated by XBP1 are interrelated in the cardiorenal system. Furthermore, XBP1 has the ability to connect mitochondria and cytoplasmic oxidative stress signals via MAMs; these signals can exert direct influence on inflammation, fibrosis, and apoptosis. On the other hand, almost all adverse changes in XBP1 expression can cause detrimental effects in cardiorenal tissues via disturbances in the pro-/antioxidant balance (Figure 4). XBP1 is not the only RNA targeted by the activity of UPR. Generally, acute injury is always accompanied by short-term and extensive ER stress, and overexpression of XBP1 may have a greater effect on promoting the production of toxic ROS. However, prolonged ER stress attenuates the expression of XBP1, and the antioxidant ability of XBP1 gradually manifests.

Given the age-dependent decline of antioxidants and the increased possibility of oxidative stress in the heart and kidneys, it is possible that the regulation of these XBP1 pathways may effectively protect the function of aging organs from oxidative damage by preserving the ER, mitochondria, RAAS, and immune homeostasis. Current treatment strategies for cardiorenal syndrome, particularly chronic diseases, are able to alleviate symptoms but have little effect on the inherent course of disease. Further exploration of the XBP1 correlative mechanisms described herein should provide valuable information for the development of novel therapeutic approaches that could improve the function of the aging cardiorenal system and guide clinical decisions relating to the management of cardiorenal syndrome.
